# Investigating associations between serum inflammatory cytokines at the time of second mild traumatic brain injury with acute neurological signs, axonal injury and behavioural outcomes in male Sprague–Dawley rats

**DOI:** 10.1093/braincomms/fcag019

**Published:** 2026-02-02

**Authors:** Justin Brand, Sandy R Shultz, David K Wright, Ashley L J J van Emmerik, Mastura Monif, Brian R Christie, Stuart J McDonald, William T O’Brien

**Affiliations:** Division of Medical Sciences, University of Victoria, Victoria, BC V8P 5C2, Canada; Division of Medical Sciences, University of Victoria, Victoria, BC V8P 5C2, Canada; Department of Neuroscience, Monash University, Melbourne, VIC 3004, Australia; Department of Neurology, The Alfred Hospital, Melbourne, VIC 3004, Australia; Centre for Trauma and Mental Health Research, Vancouver Island University, Nanaimo, BC V9R 5S5, Canada; Department of Neuroscience, Monash University, Melbourne, VIC 3004, Australia; Department of Neuroscience, Monash University, Melbourne, VIC 3004, Australia; Department of Neuroscience, Monash University, Melbourne, VIC 3004, Australia; Department of Neurology, The Alfred Hospital, Melbourne, VIC 3004, Australia; Division of Medical Sciences, University of Victoria, Victoria, BC V8P 5C2, Canada; Island Medical Program, University of British Columbia, Victoria, BC V8P 5C2, Canada; Department of Psychology, San Diego State University, San Diego, CA 92182, USA; Department of Neuroscience, Monash University, Melbourne, VIC 3004, Australia; Department of Neurology, The Alfred Hospital, Melbourne, VIC 3004, Australia; Department of Neuroscience, Monash University, Melbourne, VIC 3004, Australia

**Keywords:** biomarkers, inflammation, neurofilament light, diffusion tensor imaging, interleukin-6

## Abstract

Mild traumatic brain injury is a risk factor to sustaining future mild traumatic brain injuries and increased symptom severity and duration following a second mild traumatic brain injury. Inflammation during neurobiological recovery is hypothesized to influence susceptibility to poorer outcomes after repetitive mild traumatic brain injury. Here, we investigated whether the inflammatory response during neurobiological recovery is related to susceptibility to increased functional and biological deficits following re-injury. To investigate this, we collected serum 1, 3, 7 or 14 days after mild traumatic brain injury in male Sprague–Dawley rats and measured levels of circulating inflammatory cytokines using the MesoScale Discovery MESO QuickPlex SQ 120MM platform to quantify interferon-gamma, interleukin-1-beta, interleukin-4, interleukin-5, interleukin-6, interleukin-10, interleukin-13, keratinocyte chemoattractant/human growth-related oncogene and tumour necrosis factor-alpha. Immediately following this blood collection, rats were given a second mild traumatic brain injury to assess associations between cytokine levels at time of second mild traumatic brain injury with behavioural outcomes, neurofilament light levels, and ex vivo diffusion tensor imaging in the 28 days following second injury. After a single mild traumatic brain injury, interleukin-10, interleukin-13, interleukin-4 and tumour necrosis factor-alpha were elevated 3 days post-injury while interleukin-10 and tumour necrosis factor-alpha levels were elevated 14-days post-injury. Furthermore, higher levels of interleukin-6 and interleukin-13 at the time of a second mild traumatic brain injury were associated with a reduced number of acute neurological signs of mild traumatic brain injury following the second injury. There were no other significant correlations between circulating cytokine levels and post-injury outcomes following correction for multiple comparisons. These findings provide initial, hypothesis-generating evidence that higher levels of circulating inflammatory cytokines at the time of a second mild traumatic brain injury may be associated with decreased susceptibility to a second mild traumatic brain injury, highlighting the complex role of inflammation in repeated mild traumatic brain injury.

## Teaser

Brand *et al.* report that levels of inflammatory cytokines at the time of a second mild traumatic brain injury are associated with signs of injury severity following the second mild traumatic brain injury. The findings offer new insights into the relationship between inflammation and susceptibility to repeated brain injury.

## Graphical Abstract

**Graphical Abstract:**
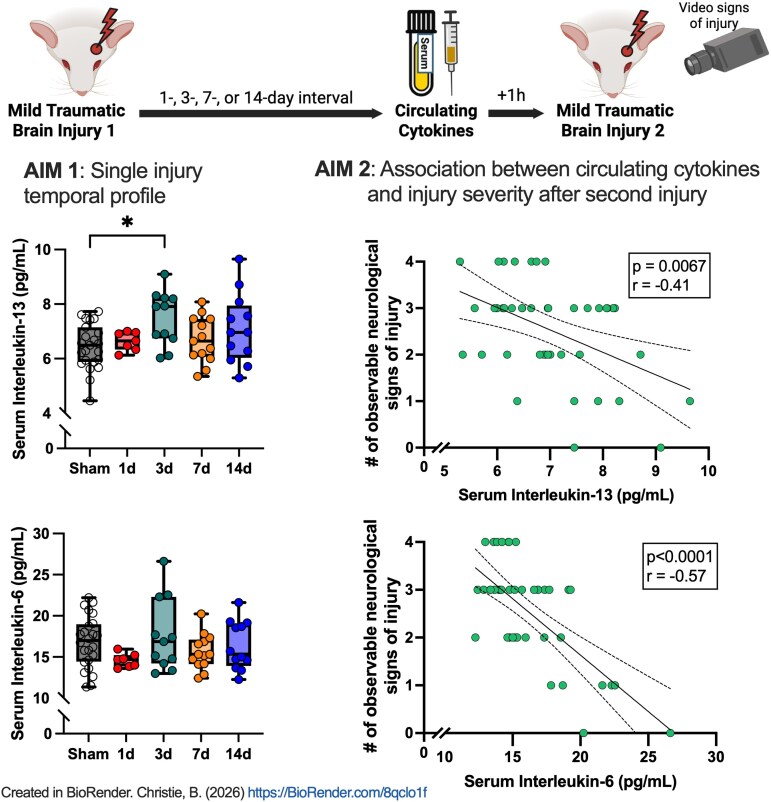

## Introduction

Traumatic brain injury (TBI) is a public health problem with more than 50 million TBIs occurring annually^1^ and the most common form being mild TBI (mTBI; also known as concussion) occurring in settings such as, motor vehicle accidents, assaults, military combat and collision sports among others.^2,3^ TBI is characterized by primary injury mechanisms consisting of mechanical shearing and tearing forces at the time of injury^1^ and by secondary injury mechanisms such as inflammation that can persist long after primary injury.^4^ Pre-clinical and clinical studies of mTBI have found that a single mTBI is sufficient to produce persistent deficits^5,6^ but a second mTBI may increase symptom severity and duration.^7,8^ After mTBI there is a hypothesized ‘window of increased cerebral vulnerability’ where ongoing neurobiological recovery and related secondary injury mechanisms can persist beyond symptom resolution.^9^ We recently investigated this hypothesis using a rat model of repetitive mTBI (rmTBI) and found that high serum levels of neurofilament light chain (NfL; a marker of neuroaxonal damage) at the time of a second mTBI is associated with greater susceptibility to a second mTBI.^10^ However, the pathophysiological mechanisms that drive the relationship between ongoing axonal injury and vulnerability to a second mTBI remain unknown.

Shortly after TBI, inflammatory responses are triggered and various cell types are recruited to the site of injury to clear debris and repair damaged tissue.^11^ Cellular changes to microglia and astrocytes promote an increase in the levels of various cytokines (e.g. TNF-α, IL-1β, IL-6, IL-10)^12-14^ while immune cells (e.g. neutrophils, monocytes, macrophages and T-cells) are recruited through a disrupted blood brain barrier^4,15-17^ that if unregulated, promotes ongoing inflammation and interferes with repair mechanisms. This can ultimately lead to neuronal damage and a variety of neurological symptoms/signs^13,18-20^. Although this shift to an inflammatory state is plastic,^21^ a persistent proinflammatory cytokine release appears to be a key mediator in TBI pathological progression.^22^

Inflammation is implicated in the pathological cascade following mTBI,^23^ however, how ongoing inflammation influences susceptibility to a second mTBI is unknown, and difficult to model in the clinical setting. Here, we have the overall goal of identifying inflammatory targets that may play a role in regulating TBI pathological progression to inform future TBI studies investigating diagnosis, prognosis and treatment strategies for rmTBI. Therefore, in this exploratory study, we utilized a rat model of mTBI and quantified a panel of circulating inflammatory cytokines at 1, 3, 7 or 14 days after a single mTBI to first determine the temporal profile of circulating inflammatory cytokines after a single mTBI. Rats were then given a second mTBI immediately after this blood collection to subsequently determine how the inflammatory response is related to susceptibility to injury, including neurological signs of injury, serum NfL levels, behavioural outcomes and diffusion tensor imaging (DTI) metrics following a second mTBI. While not a direct measure of neuroinflammation, circulating cytokines correlate with neuroinflammation regulation and neurodegenerative disorder progression, and provide an accessible and translatable measure of neuroinflammation.^24,25^ We hypothesized that the greatest inflammatory response would be seen one day following a single injury and that rats with elevated circulating inflammatory markers at the time of a subsequent mTBI would have greater behavioural deficits, increased neuroaxonal damage, and decreased white matter integrity.

## Materials and methods

This study represents a secondary analysis of an existing experimental cohort that was previously described in detail.^10^ For the previous study, adolescent male Sprague–Dawley rats underwent sham, single mTBI, or repeated mTBI procedures with outcomes including neurological signs of injury, serum NfL, behavioural testing, and DTI reported. In this follow up study, we performed new analyses of serum collected from this cohort to quantify inflammatory cytokines levels at the time of a second injury, and re-analysed the previously acquired outcomes to test their association with cytokine levels. All analyses are newly conducted, and no results, figures or statistical outputs are duplicated from the previous publication. The primary outcome of the study was to assess the association between serum inflammatory cytokine levels at the time of second injury with observable neurological signs of injury and 3-day NfL levels, as these are objective outcomes that we have previously shown to be robustly changed following mTBI.^10,26^

### Animals

As previously reported,^10^ 84 male adolescent Sprague–Dawley rats were obtained from the Monash Animal Research Platform (Clayton, Victoria, Australia). Male rats were used in this exploratory study to minimize the number of groups, and biological variability that may obscure small effect sizes. Rats were housed in cages of three on a 12-h light/dark cycle with food and water available ad libitum. All procedures were approved by the Alfred Medical Research and Educational Precinct Animal Ethics Committee (E/2081/2021/M). Animal studies are reported in compliance with the ARRIVE guidelines and the Australian code of practice for the care and use of animals for scientific purposes by the Australian National Health and Medical Research Council.

### Experimental groups

All group assignments and randomization were conducted previously^10^; no new randomization was performed for the analyses reported here. Briefly, in the original study, rats were randomly assigned to the sham group (two sham procedures), a single mTBI group (sham procedure followed by an mTBI procedure) or the repeat mTBI group (rmTBI; two mTBI procedures). The rmTBI rats were then divided into inter-injury intervals of 1, 3, 7 or 14 days. Experimental cohorts were randomly allocated to have a consistent day of first mTBI/sham (PND37-56) or second mTBI/sham (PND43-57) to achieve a consistent mean postnatal day (PND 47) of injury/sham for all groups. As described previously, 16 rats were excluded from final analyses. Six rats were deemed to have undergone an unsuccessful mTBI procedure (i.e. underwent an mTBI that was deemed unsuccessful by an investigator at time of procedure, and recorded a score of zero by a second blinded investigator on video analysis of observable neurological signs of mTBI as described previously^10^), one died immediately following mTBI procedure, one had a cage-associated injury, and eight had insufficient blood collections. Therefore, final group numbers included in this convenience sample analyses are rmTBI-1d (i.e. 1d interval, *n* = 7), rmTBI-3d (*n* = 11), rmTBI-7d (*n* = 13), rmTBI-14d (*n* = 12), single mTBI (*n* = 12), and sham (*n* = 12).

**Figure 1:**
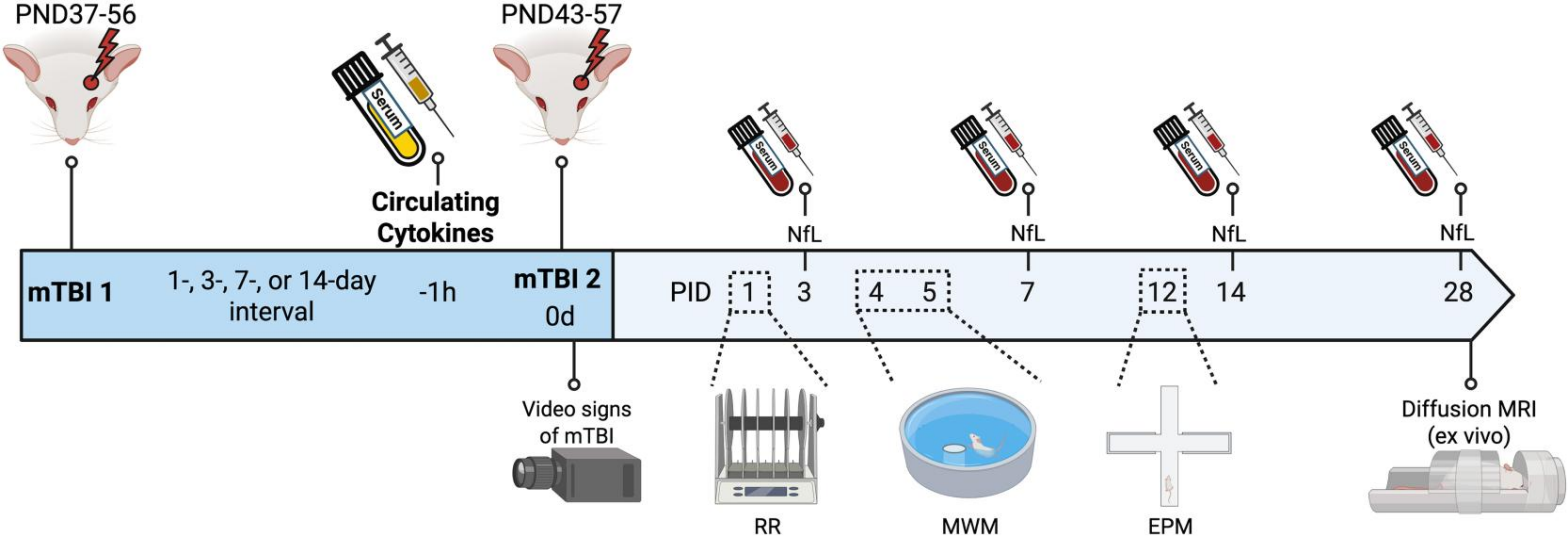
**Experimental design timeline.** Rats were subjected to two mTBI’s separated by varying inter-injury interval (or sham equivalent). Blood was collected immediately prior to second injury to quantify circulating inflammatory response following a single injury and then determine the relationship with neuroaxonal damage, behavioural deficits, and chronic white matte integrity after a second injury. Serum cytokine levels are newly generated for this study with all other primary data collected previously.^10^ Experimental cohorts were randomly allocated to have a consistent day of first mTBI/sham (PND37-56) or second mTBI/sham (PND43-57) to achieve a consistent mean postnatal day (PND47) of injury/sham for all groups. PND = post-natal day, PID = post-injury day, relative to second sham/mTBI procedure, RR = Rotarod, MWM = Morris Water Maze, EPM = Elevated-plus maze. Figure created in BioRender: Christie, B. (2026) https://BioRender.com/ke743ww.

To assess the inflammatory response after a single mTBI and the relationship between these levels at the time of a second injury with subsequent outcomes, blood was collected immediately prior to the second mTBI/sham procedure (i.e. 1, 3, 7 or 14 days post the first mTBI/sham). For Aim 1, sham and single mTBI groups were combined into a single sham group as the single mTBI group was yet to receive an mTBI (*n* = 24). For Aim 2, following the second mTBI, all rmTBI groups (*n* = 43) were collapsed irrespective of inter-injury interval. See Fig. 1 for experimental design timeline.

With the a priori hypothesis that there would be temporal changes in serum cytokines following a single mTBI, this approach was taken to generate a broad biological range of cytokine levels at the time of re-injury, allowing investigation of their association with observable neurological signs of injury, neuroaxonal damage, behavioural outcomes and DTI parameters. Although time between injury is a key factor that contributes to neurobiological disruption and recovery, other factors such as differences in previous injury severity, anatomy, and post-injury environment may differ between individuals.^10^ Therefore, in this study, cytokines were examined as a biological indicator for susceptibility to a second mTBI irrespective of inter-injury interval.

### Awake closed head injury model of mild traumatic brain injury

The ACHI model is a well characterized and clinically relevant model of concussive-like injury^10,26,29-31^. The mTBI or sham protocol was followed as previously described.^10,27^ For the mTBI/sham procedure, animals were place in the restraint cone, fitted with a 3D printed steel helmet with an impact site over the left parietal bone and immobilized on a foam platform. The impactor was then fired with a velocity of 6.5 m/s, an extension depth of 10 mm and dwell time of 100 ms. Sham animals underwent an identical procedure, but the impactor was not fired.

### Video quantification of observable neurological signs of mild traumatic brain injury

Video quantification of four observable neurological signs of mTBI were performed, immediately after each mTBI, as previously described.^10^ This four-point scale was developed based on the ‘International consensus definitions of video signs of concussion’^30^ and quantifies the presence of (i) postimpact seizures/tonic posturing, (ii) motionlessness, (iii) forelimb incoordination, where one or both forelimbs were unable to grasp a 2 cm beam and (iv) hindlimb incoordination, where the hind limbs slip off the 2 cm beam and/or the rat was unable to balance on the beam. Presence of an observable sign was given a score of one for each parameter. Videos were analysed by a single researcher blinded to the experimental conditions.

### Behaviour

Following the final mTBI/sham procedure, all animals underwent a behavioural testing paradigm. Researchers were blinded to group allocation for all behavioural testing and subsequent experimental analyses. All behaviour tests were scored by a single researcher as per previous study.^10^ Note: The results for this behavioural testing battery, serum NfL levels, and DTI metrics are included for the rmTBI rats only to investigate the relationship between levels of inflammatory cytokines at the time of a second mTBI with subsequent outcomes. The performance of the sham and single mTBI, and the effect of inter-injury interval on these outcomes has been previously described.^10^

### Rotarod

Sensorimotor function was assessed using the rotarod task at baseline (15 days prior to the second mTBI) and 1-day post final mTBI/sham. As previously described, rats were placed on a rod accelerating from 4 to 40 rpm over a five-minute period. Three trials were performed at each timepoint with latency quantified. The task was analysed as 1-day latency to fall relative to baseline latency. The task was also performed at 2, 6, 13 and 27 days post final mTBI/sham with this data presented previously.^10^

### Morris water maze

As previously described, rats underwent a 2-day water maze protocol to assess spatial memory.^28^ A 175 cm diameter pool was filled with warm water (28°C) with an escape platform located 3.5 cm below water level and four distinct visual cues placed above the edge of the pool. Rats underwent 10 trials at 4 days (acquisition testing) and 5 days (reversal testing) after final mTBI/sham. Latency to find the platform was averaged across the 10 trials for the respective testing days. Rats were tracked by TopScanLite Version 2.0.

### Elevated plus maze

The EPM was performed to measure anxiety-like behaviour as previously described.^27,28^ The maze is a ‘plus-shape’ and contains two arms with 30 cm high walls (i.e. closed arms) and two arms without walls (i.e. open). 12 days after final mTBI/sham procedure, rats were placed in the centre of the EPM and allowed to freely explore for 5 min with time spent in the open arms quantified using TopScanLite Version 2.0.

### Blood and tissue collection

Blood and tissue collection was performed as previously described.^10^ Rats were lightly anaesthetized via isoflurane inhalation where whole blood was collected into BD SST microtainer tubes by inserting a 23” gauge needle into the lateral tail vein 1 h prior to final mTBI/sham (i.e. 1, 3, 7 or 14 days after the first mTBI). Blood was centrifuged at 1500 g for 10 min then serum collected and stored at −80°C. This process was repeated 3-, 7-, and 14 days post final mTBI/sham. At 28-days post final mTBI/sham rats were euthanized as previously described^28^ to collect whole blood via cardiac puncture and brains fixed in paraformaldehyde. Note: 3-, 7-, 14- and 28-day blood samples were analysed for serum NfL only.

### Serum inflammatory cytokine quantification

Inflammatory cytokines interferon-gamma (IFN- ), interleukin-1-beta (IL-1 *β*), interleukin-4 (IL-4), interleukin-5 (IL-5), interleukin-6 (IL-6), interleukin-10 (IL-10), interleukin-13 (IL-13), keratinocyte chemoattractant/human growth-*γ*oncogene (KC/GRO) and tumour necrosis factor-alpha (TNF-α) were quantified on a MESO QuickPlex SQ 120MM (Meso Scale Discovery®, Rockville, MD, USA) using Meso Scale Discovery (MSD) V-PLEX Proinflammatory Panel 2 Rat Kits (Meso Scale Discovery, Inc) per manufacturer’s instructions. All samples were run in duplicate. All data was analysed using Discovery Workbench 4.0 software (Meso Scale Discovery, Rockville, MD, USA). Inclusion criteria were more than 75% of samples within in detection range with duplicate coefficient of variance (CV) less than 25%. The assays’ lower limits of detection (LLOD), intraplate coefficient of variance, and interplate coefficient of variance are outlined in Supplementary Table 1. All samples were in detection range for IL-4, IL-6, IL-10, IL-13, KC/GRO and TNF-α. Out of 67 total samples ran, IL-1 *β* was undetectable in 59 samples (88% undetected) and IL-5 was undetectable in 62 samples (93% undetected). As such, all IL-1*β* and IL-5 results were excluded from all analyses. A total of 15 samples were below the LLOD for IFN-*γ* and were assigned the LLOD for the respective plate.

### Serum neurofilament light quantification

NfL was quantified as previously described.^10^ Briefly, Simoa NfL Advantage Kits (Quanterix, Billerica, MA) were used on a SIMOA HD-X Analyzer following the manufacturer’s instructions. All samples were above the lower limit of detection (0.038 pg/mL) and measured in duplicate with an average duplicate CV of 6.2%.

### Magnetic resonance imaging

DTI was performed as previously described.^10^ Briefly, a 9.4 T Bruker MRI was used to acquire a two-shot echo planar imagine sequence with diffusion-weighting performed in 61 directions with δ = 4.2 ms, Δ = 12 ms and b-values = 2000 and 4000 s/mm^2^. Two b = 0 (b_0_) volumes were acquired and other imagine parameters were adjusted to give an isotropic resolution of 250 μm: repetition time = 8 s; echo time = 50 ms; field of view = 3.2 × 2.4 cm^2^; and 48 axial slices. Distortion correction using FSL’s topup was attained by acquiring a subsequent b_0_ image with the same imaging parameters and the phase reversed.^31^ Image processing was performed as previously described using FSL and MRtrix3 software.^32,33^ Mean fractional anisotropy (FA), apparent diffusion coefficient (ADC), radial diffusivity (RD) and axial diffusivity (AD) values were obtained for ipsilateral (left hemisphere, LH; site of injury) and contralateral (right hemisphere, RH) for the corpus callosum, external capsule, internal capsule, and fimbria.

### Statistics

Circulating cytokine levels 1, 3, 7 and 14 days after the first injury were analysed for outliers using the ROUT method with *Q* = 5% which identified one sham IL-13, one sham KC/GRO, two 7 day KC/GRO and three sham TNF-α samples as outliers. Normality of cytokine levels were then analysed using the Shapiro–Wilks test. A one-way analysis of variance (ANOVA) or Kruskal–Wallis was used where appropriate to investigate group differences in cytokine levels between the sham group and rats 1, 3, 7 or 14 days after a single mTBI. Sample size for each cytokine based on group are sham (*n* = 19–24), 1 day (*n* = 4–7), 3 days (*n* = 10–11), 7 days (*n* = 10–13) and 14 days (*n* = 11–12). Exact sample size based on cytokine and group is reported in Supplementary Table 2. Post-hoc analyses were performed with Bonferroni’s multiple comparisons for parametric data or Dunn’s method for non-parametric data relative to the sham group. The rmTBI groups were then collapsed irrespective of inter-injury interval to obtain a variance of cytokine levels prior to a second mTBI. Cytokine levels irrespective of inter-injury interval were then assessed for their association to subsequent outcomes. Normality of cytokine levels, observable neurological signs of injury, serum NfL levels (Ln transformed), behavioural outcomes and all DTI parameters for each ROI were assessed using the Shapiro–Wilks test. Spearman’s or Pearson’s correlations where appropriate assessed for the association of cytokines at the time of a second mTBI with observable neurological signs of injury, Ln transformed serum NfL, rotarod, water maze, EPM and DTI measures. Data from all 67 rats were included in analyses of the relationship between levels of inflammatory cytokines with all post-injury outcomes. Analyses were two tailed with multiple comparison corrections performed. For the correlation between serum cytokine levels with observable neurological signs of injury, serum NfL levels, and behavioural outcomes an adjusted *α*-value of 0.0071 was used for each outcome to account for comparison to the seven cytokines. For analyses testing the association between serum cytokines and DTI measures an adjusted *α*-value of 0.0009 was used to account for the analyses between the seven cytokines and eight brain regions. Analyses were also performed within the sham group with no significant correlations found (data not shown). All statistical analyses were performed with GraphPad Prism version 10.1.1 for macOS (GraphPad Software, La Jolla, California).

## Results

### Inflammatory response following a single mild traumatic brain injury

**Figure 2:**
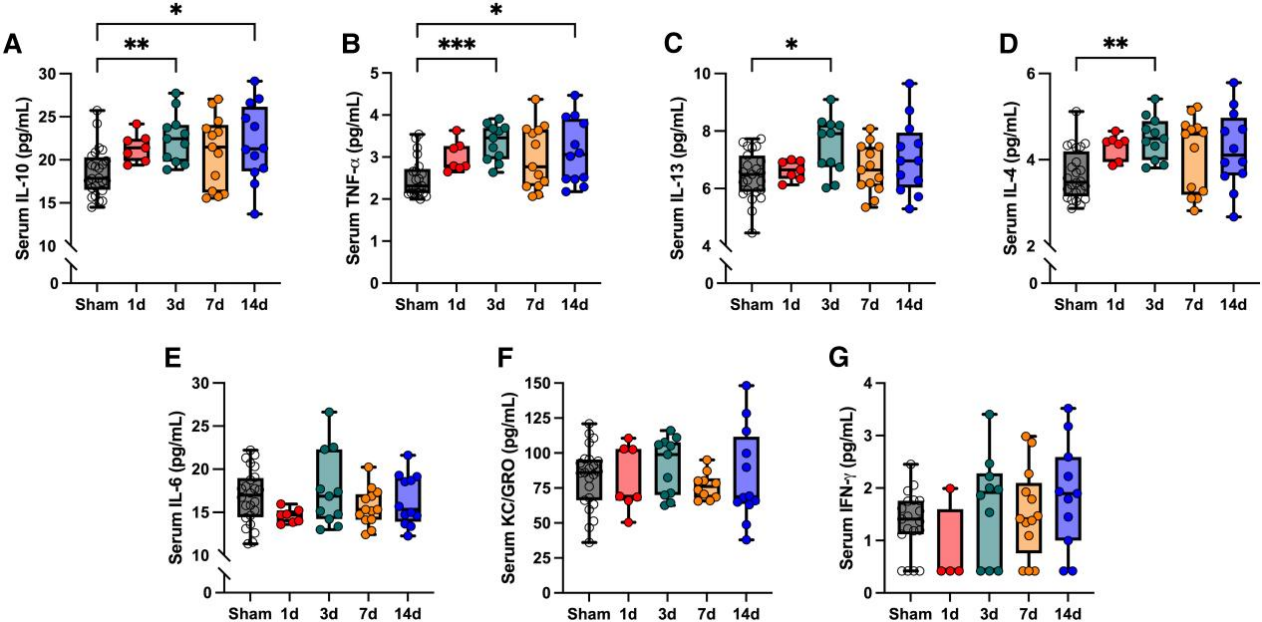
**Cytokine concentration after a single sham/mTBI procedure.** There was an effect of group for IL-10 (**A**; *F*_4,62_ = 3.66, *P* = 0.009), with levels elevated in rats 3 days (95% CI: −7.14 − 0.79, *P* = 0.008) and 14 days (95% CI: −6.44−−0.28, *P* = 0.03) post-mTBI compared to sham-injured rats. Similarly, for TNF-α (**B**), there was an effect of group (*H* = 16.65, *P* = 0.002) with levels elevated at 3 days (Median Sham = 2.32 pg/ml, Median 3d = 3.46 pg/ml, *P* = 0.0007) and 14 days (Median Sham = 2.32 pg/ml, Median 14d = 3.06 pg/ml, *P* = 0.03) post-mTBI compared to sham-injured rats. For IL-13 (**C**), there was an overall effect of group (*F*_4,61_ = 2.74, *P* = 0.04), with levels higher at 3 days post mTBI (95% CI: −1.92 − 0.17, *P* = 0.01) compared to sham-injured rats. For IL-4 (**D**), there was also an overall effect of group (*H* = 14.79, *P* = 0.005) with levels elevated 3 days post-mTBI (Median Sham = 3.48 pg/ml, Median 3 days = 4.49 pg/ml, *P* = 0.003). There was no effect of group for serum IL-6 (**E**; *F*_4,62_ = 1.39, *P* = 0.25), KC/GRO (**F**; *F*_4,58_ = 0.49, *P* = 0.74) or IFN-γ (**G**: *H* = 5.18, *P* = 0.27). Ordinary one-way ANOVAs with Bonferroni correction was used for IL-10, IL-13, IL-6 and KC/GRO. Kruskal–Wallis test with Dunn’s multiple comparison tests were used for TNF-α, IL-4, and IFN-γ. Sample sizes per group for each cytokine are: Sham (*n* = 19–24), 1 day (1d; *n* = 4–7), 3 days (3d; *n* = 10–11), 7 days (7d; *n* = 10–13) and 14 days (14d; *n* = 11–12). Individual data points are shown for all analyses. The experimental unit is a single biological replicate (i.e. one rat). *P < 0.05, **P < 0.01, ***P < 0.001; Box plots represent median, IQR.

A significant effect of group was found for IL-10 (*F*_4,62_ = 3.66, *P* = 0.009), IL-13 (*F*_4,61_ = 2.74, *P* = 0.04), IL-4 (*H* = 14.79, *P* = 0.005) and TNF-α (*H* = 16.65, *P* = 0.002). Post-hoc analysis revealed levels of IL-10 (Fig. 2A; 95% CI: −7.14 − 0.79, *P* = 0.008), TNF-α (Fig. 2B; Median Sham = 2.32 pg/ml, Median 3d = 3.46 pg/ml, *P* = 0.0007), IL-13 (Fig. 2C; 95% CI: −1.92 − 0.17, *P* = 0.01) and IL-4 (Fig. 2D; Median Sham = 3.48 pg/ml, Median 3d = 4.49 pg/ml, *P* = 0.003) were elevated 3 days post injury compared to sham. Levels of IL-10 (Fig. 2A; 95% CI: −6.44−−0.28, *P* = 0.03) and TNF-α (Fig. 2B; Median Sham = 2.32 pg/ml, Median 14 d = 3.06 pg/ml, *P* = 0.03) were also elevated 14 days post injury compared to sham. No other timepoints were different compared to sham. There were no significant effects of group for IL-6 (Fig. 2E; *F*_4,62_ = 1.39, *P* = 0.25), KC/GRO (Fig. 2F; *F*_4,58_ = 0.49, *P* = 0.74) or IFN-γ, (Fig. 2G; *H* = 5.18, *P* = 0.27).

### Serum inflammatory cytokine at the time of second mild traumatic brain injury correlate with decreased signs of injury

**Figure 3:**
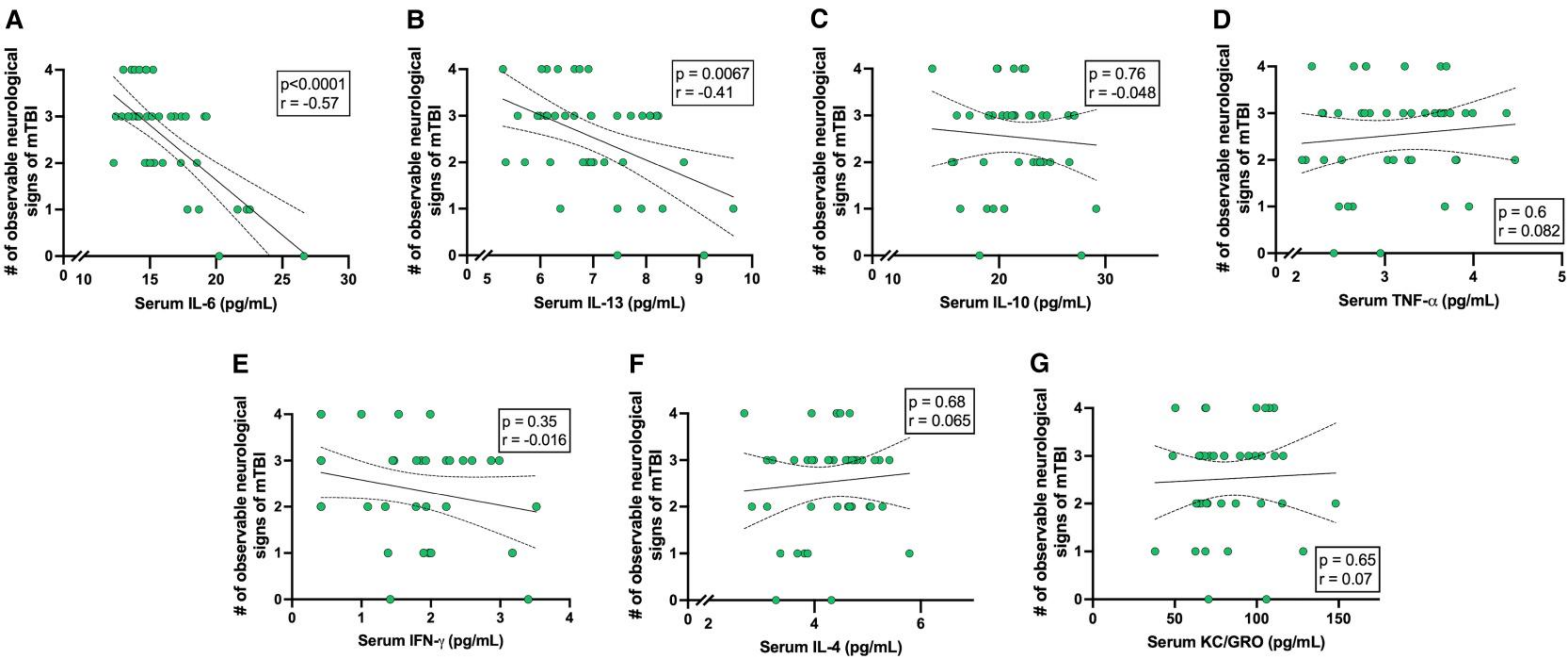
**Associations between serum circulating cytokines collected immediately prior to a second mTBI and the number of observable neurological signs of injury after the second mTBI.** Serum IL-6 (**A**: *r* = −0.57, *P* < 0.0001, *n* = 43) and IL-13 (**B**; *r* = −0.41, *P* = 0.0067, *n* = 43) negatively correlated with the number of observable neurological signs of mTBI. No significant correlations were observed for IL-10 (**C**; *r* = −0.048, *P* = 0.76, *n* = 43), TNF-α (**D**; *r* = 0.082, *P* = 0.60, *n* = 43), IFN-γ (**E**; *r* = −0.016, *P* = 0.35, *n* = 38), IL-4 (F; *r* = 0.065, *P* = 0.68, *n* = 43) or KC/GRO (**G**; *r* = 0.07, *P* = 0.65, *n* = 40). Spearman analyses were used for all analyses. Solid black line represents regression line, dashed lines represent 95% confidence bands of the regression line. Individual data points are shown for all analyses. The experimental unit is a single biological replicate (i.e. one rat).

Spearman’s correlation analyses were used to test the association between serum cytokine levels at the time of second injury and the number of observable neurological signs of injury following the second mTBI. Observable neurological signs negatively correlated with IL-6 (Fig. 3A; *r* = −0.57, *P* < 0.0001) and IL-13 (Fig. 3B; *r* = −0.41, *P* = 0.0067). These associations remained significant after correction for multiple comparisons (adjusted *α*-value of 0.0071). No other correlations between cytokines and observable neurological signs of injury were found prior to, or following correction for multiple comparison (Supplementary Table 3).

### Associations between serum inflammatory cytokine levels and subacute behavioural outcomes

Spearman’s correlations were used to assess the relationship between inflammatory cytokine levels at the time of second injury and subacute behavioural tasks. Prior to correcting for multiple comparisons, there was an association between IL-4 and average latency to find platform in the water maze acquisition testing (*r* = 0.32, *P* = 0.04); however, this did not survive correction for multiple comparisons. There were no significant correlations between serum inflammatory cytokine levels and behavioural tasks prior to or following multiple comparison correction (Supplementary Table 4). Furthermore, there were no significant correlations between serum inflammatory cytokines with any behavioural tasks within the sham group (data not shown).

### Inflammatory cytokines and neurofilament light associations

Spearman’s correlations analyses tested the association between inflammatory cytokine levels at the time of second mTBI and serum NfL levels at time of second mTBI and 3, 7, 14 and 28 days post second mTBI. Prior to correcting for multiple comparisons, there was an association between IFN-γ and NfL levels at 3 days (*r* = −0.35, *P* = 0.03) and 7 days (*r* = −0.39, *P* = 0.02) post-second mTBI and IL-6 levels associated with NfL at 3 days (*r* = −0.35, *P* = 0.02) post second mTBI. After correcting for multiple comparisons, there were no significant correlations between serum inflammatory cytokine levels and serum NfL levels at any timepoint. Full statistical comparisons for repetitive mTBI group provided in Supplementary Table 5. No significant correlations were found between serum inflammatory cytokines and serum NfL at any timepoint within the sham group (data not shown).

### Associations between inflammatory cytokine levels and chronic white matter integrity

Pearson’s or Spearman’s correlation analyses were used where appropriate to investigate the relationship between inflammatory cytokine levels at the time of second mTBI with DTI metrics (FA, ADC, RD and AD) in the left and right corpus callosum, internal capsule, external capsule, and fimbria. Prior to correction for multiple comparisons, diffuse associations were observed between IL-6 levels at the time of second mTBI with ADC, RD and AD 28 days post second mTBI. Additional, but fewer, correlations were also found between IFN-γ and IL-13 with AD in the right fimbria as well as KC/GRO with ADC, RD and ADC in the left and right internal capsule (see Supplementary Tables 6 and 7 for full results). However, no significant correlations remained following correction for multiple comparisons (adjusted *α*-value of 0.0009). Prior to correcting for multiple comparisons, there were associations observed between KC/GRO and ADC, RD and AD within the sham group; however, no associations survived multiple comparison correction (data not shown).

## Discussion

In the aftermath of mTBI, secondary injury mechanisms such as inflammation may leave the brain in a state of increased vulnerability to a second mTBI.^7,8^ Fluid biomarkers released following mTBI have the potential to characterize injury severity, measure pathophysiological disruption and recovery, as well as predict subsequent outcomes.^34^ This study investigated circulating measures of inflammation, a critical component of the pathophysiological response to mTBI, to determine the relationship between inflammation at the time of a second mTBI with observable neurological signs of injury severity, neuroaxonal damage, subacute behavioural and chronic white matter integrity deficits after a second mTBI.

We found that the peripheral cytokine response to a single mTBI peaked 3 days after injury with levels of IL-10, IL-13, IL-4 and TNF-α significantly elevated compared to sham. Levels of IL-10 and TNF-α were further elevated 14 days after mTBI. Subsequently, by delivering a second mTBI immediately after this blood collection, we found a fair, negative correlation between the levels of inflammatory cytokines IL-6 and IL-13 with the number of observable neurological signs, suggesting a potential association with reduced acute injury severity. Furthermore, while preliminary negative correlations were observed between IFN-γ and IL-6 with post-injury NfL levels, and diffuse associations between IL-6, IFN-γ, IL-13 and KC/GRO with DTI metrics 28 days post second mTBI, these associations did not survive multiple comparison corrections. Together, however, these findings provide novel evidence that the inflammatory response at the time of a second mTBI may be associated with susceptibility to rmTBI.

### Temporal changes in serum inflammatory markers after a single mild traumatic brain injury

The inflammatory response following TBI is a complex interaction that can be beneficial by promoting clearance of debris and regeneration, whilst also having the potential to become detrimental should this immune response become dysregulated or excessive.^11,35^ Here, we first assessed the temporal profile of circulating cytokines after a single mTBI, where we found the greatest increase of inflammatory cytokines 3 days after injury, contrary to our hypothesis. The significant increases we found 3 days post mTBI corroborate previous studies that found increases in cytokines 3 days after mTBI in rats^36-38^. Similarly, in the brain homogenates of male mice IL-10 was shown to be elevated 3 days post injury compared to sham.^39^ Interestingly, while we did not find significant differences in any cytokine 7 days after mTBI, IL-10 and TNF-α were significantly elevated 14 days post injury. This may reflect a potential glial cell shift to a chronic inflammatory phenotype.^13^ Similar to our findings, gene expression of both pro- and anti-inflammatory cytokines (including IL-10) have previously been shown to increase 14 days after TBI, with the elevation of some cytokines persisting for at least 60 days after TBI.^40^ Future studies are required to determine whether the elevation of IL-10 and TNF-α found remain chronically elevated.

It is important to note that here we investigated circulating cytokines opposed to neuroinflammation specific to the central nervous system (CNS). Although direct measures of inflammation in brain tissue immediately prior to a second mTBI are a more accurate indicator of neuroinflammation, it is not feasible to investigate this in vivo immediately prior to a mTBI without causing considerable confounding neurobiological disruption. Furthermore, circulating inflammatory biomarkers have been shown to correlate to CNS measures of neuroinflammation and maintain translatable potential to the clinical setting.^41,42^

### Inflammatory response association with subsequent outcomes following re-injury

Here, we assessed the relationship between levels of inflammatory cytokines prior to a second injury and the susceptibility to neurological signs of injury severity, neuroaxonal injury, behavioural deficits, and chronic white matter integrity deficits after a second injury. We found that higher levels of serum IL-6 and IL-13 at the time of second injury was associated with fewer observable signs of mTBI following a second mTBI. We have recently shown that the presence of observable neurological signs, such as loss of consciousness, is associated with more substantial and prolonged neurobiological changes after mTBI.^43^ Interestingly, while IL-6 concentrations were not significantly elevated after a single injury compared to sham, there was an association between IL-6 levels and acute observable signs of injury. IL-6 is a pleiotropic cytokine with both detrimental effects by promoting the acute phase inflammatory response following TBI while also having the ability to provide beneficial effects such as promoting neuronal differentiation, inhibition of TNF-α synthesis, and promoting nerve growth factor synthesis.^34^ This dual role may explain why higher circulating IL-6 levels at the time of injury were linked to fewer observable signs of injury in the present study, with pre-existing elevations in IL-6 potentially engaging protective pathways that attenuate susceptibility to mTBI. In contrast, IL-13 was significantly elevated 3 days after a single injury compared to sham, and levels of IL-13 were associated with the number of acute observable signs of injury. IL-13 is an anti-inflammatory cytokine that modulates microglia^44,45^ and macrophage activation.^46,47^ Post-injury administration of IL-13 has been shown to improve functional recovery, reduce neuronal tissue loss and reduce the number of proinflammatory microglia after TBI^48^ and stroke.^49^ Therefore, increased levels of IL-13 prior to a second injury may modulate inflammatory cascades and therefore decrease susceptibility to re-injury following mTBI. However, given these findings are correlational, future work should focus on elucidating the mechanism underpinning this relationship, including whether this relationship is specific to a rmTBI, or if it remains in the absence of a previous mTBI, and whether other biological process such as stress or unrelated inflammatory process, may underpin the relationship.

Furthermore, although not passing multiple comparison corrections, further non-significant negative correlations were seen between IL-6 with 3-day serum NfL levels and diffuse correlations between serum IL-6 with DTI metrics 28 days following the second mTBI. NfL is a highly sensitive and specific biomarker to neuroaxonal damage while DTI metrics provide insights to microstructural tissue properties of white matter.^50^ By including these two outcome measures, our work suggests the inflammatory response may be related to neuroaxonal damage and chronic white matter integrity. Interestingly, reduced ADC and AD has been observed in concussed athletes^51-54^ suggesting that positive correlations with these metrics may influence the maintenance of white matter integrity. Interpretation of DTI metrics remains challenging as other pathological mechanisms such as oedema and demyelination may affect measurements following TBI.^55^ However, as NfL levels have consistently been shown to be elevated after mTBI in humans^42^ and rats,^28^ coupling NfL and DTI metrics allow us to speculate that the trends we find between circulating inflammatory cytokines, NfL, and DTI metrics may provide preliminary evidence that a greater inflammatory response immediately prior to a second injury may protect white matter integrity following a subsequent injury. It must be noted, however, that these findings did not survive multiple comparison corrections, and as such significant future investigations are required to determine whether the inflammatory response at the time of re-injury is related to white matter integrity preservation following rmTBI. Furthermore, while this study focused on univariate associations, the cytokine response to mTBI is likely complex and interconnected. Future work in larger cohorts may benefit from multivariate analyses, such as Principal Component Analysis, to identify the profiles of cytokine expression that may collectively be indicative of injury susceptibility.

### The role of inflammation in the neurobiology of ‘recovery’

The clinical recovery from mTBI is currently defined by its symptom presentation, yet we know this does not mirror neurobiological recovery^51,56-58^ and that time between injuries may be one critical factor influencing outcomes.^59,60^ After TBI, the inflammatory response is highly dynamic with various mechanisms acting at different timepoints. In humans, cytokine release coupled with immune cell infiltration typically peak between 24 and 72 h after injury and microglial shifts to a diseased state peaks 7 days post injury.^11,61^ Thus, this 24-h to 7-day window may be of greatest concern. Pre-clinically, injuries sustained closer in time show exacerbated glial fibrillary acidic protein, NfL and Tau expression,^62^ microglial activation and neuroinflammation 8 + weeks post injuries,^63^ greater axonal degradation^51,64^ and exacerbated inflammation.^65^ A greater number of injuries have also been associated with exacerbated inflammation.^23^ Although the interval between injuries and the number of injuries is undoubtedly related to outcomes from rmTBI, we hypothesize that an individual’s state of neurobiological recovery, which may be influenced by multiple factors including age at injury, previous injury severity, sex, prior concussion history as well as inter-injury interval, drives this relationship.

In our previous study, we found no relationship between inter-injury interval on post-injury outcomes but found that high levels of NfL at the time of a second mTBI was related to a greater potentiation of NfL levels and more neurological signs of injury after a second mTBI. Furthermore, we found that this relationship persisted even when the analysis was stratified to only include rats with a 7- or 14-day inter-injury interval.^10^ Similarly, a previous study found that one injury every day for 5 days caused progressive neurological deficits, however when classified as susceptible or resilient to rmTBI, the resilient mice were similar to controls across all parameters tested yet susceptible mice showed exacerbated deficits.^66^

In this study, we found that levels of IL-6 and IL-13 at the time of a second mTBI were related to the number of observable neurological signs following the injury. While collapsing the inter-injury interval groups does not allow insight into the temporal dynamics of this relationship or the duration of cerebral vulnerability, it provides further evidence that the neurobiological state at the time of a second mTBI may be associated with susceptibility to re-injury. This may be particularly pertinent in settings where there is an increased risk of repeated mTBI exposure such as violent intimate partner relationships,^67^ contact sports,^68^ and military deployments.^69^ It should be noted, however, that the developmental window may contribute to susceptibility to mTBI and prolonged neurobiological recovery. For example, mTBIs sustained in adolescence, as used in this study, can alter on-going brain development,^70^ with pre-clinical adolescent models showing that compared to adults, adolescents show distinct patterns of functional deficits including worse spatial memory and no long-term reduction in white matter area.^71^ Furthermore, injuries sustained in adolescence may also alter microglial function and their role in chronic neuroinflammation.^72^ Given the potential increased susceptibility to poorer outcomes from mTBIs sustained during adolescence, future studies are required to determine whether the current findings translate to other developmental windows.

While requiring further investigations including determining the temporal dynamics and biomarker thresholds for increased susceptibility, objective measures of the neurobiological state following mTBI could enhance understanding of recovery trajectories and ultimately support neurobiologically informed guidelines regarding safe return to play, duty and other high-risk activity decisions.

### Limitations

Although this work provides novel insight into the temporal profile of circulating inflammatory response after a single injury and how circulating inflammatory cytokines are associated with subsequent outcomes, there are limitations that should be considered. Firstly, the current study only investigated male rats which may limit the generalizability of our findings to females. Females have been reported to experience more cognitive and emotional symptoms compared to males while also experiencing longer symptom duration.^73-75^ Known sex differences in the immune response,^76^ inflammation,^77^ TBI pathology^72,78^ and inflammation following TBI,^79-82^ may contribute to the differential symptom presentation and duration. Specifically, males with a history of concussion have elevated levels of serum IL-1β compared to females with history of concussion, while females with no history of concussion are not different from those with a history of concussion.^81^ Furthermore, mRNA TNF-α expression is significantly elevated at 4 h post injury in females compared to males.^83^ Females also showed higher levels of granulocyte colony-stimulating factor (G-CSF) and monocyte chemoattractant protein-1 in serum as well as IL-10, G-CSF, interferon gamma-induced protein-10, and mouse keratinocyte-derived chemokine in whole brain samples compared to males.^84^ Given the commonly reported sex differences in the inflammatory response to mTBI, future studies are required to determine whether the interaction between cytokine levels and mTBI susceptibility may differ in females.

Additionally, we investigated circulating inflammatory cytokines in the periphery. While no studies using the ACHI model have directly examined associations between circulating cytokines with measures of neuroinflammation, previous studies in other TBI models have found a relationship. For example, plasma cytokine levels have been found to correlate with cerebellar cytokine expression in a paediatric weight drop model of mTBI in rats.^85^ Furthermore, levels of plasma cytokines including IL-6, IL-1β and TNF-α have been shown to correlate with higher white matter integrity in a controlled cortical impact model of TBI.^86^ Although these findings suggest that circulating cytokines may provide insights into neuroinflammation and CNS injury, it should be considered that the current study did not directly assess central measures of inflammation.

Furthermore, whilst collapsing the rmTBI groups allowed us to investigate how markers of inflammation were related to susceptibility to a second injury, independent of inter-injury interval, we were not able to quantify the relative contribution of time post-injury versus ongoing neurobiological recovery alone. Finally, it is important to note that the findings that IL-6 and IL-13 are associated with reduced observable neurological signs are correlational, and future studies are required to determine whether IL-6 and IL-13 contribute causally to rmTBI susceptibility.

## Conclusion

Here, we found that male rats given an mTBI had an extended inflammatory response, as measured by circulating cytokine levels, with individual cytokines elevated at 3 and 14 days after a single injury. Furthermore, we found that serum IL-6 and IL-13 at the time of a second mTBI negatively correlated with the number of observable neurological signs immediately after a second injury. Although future studies are required to elucidate the relative contribution of time-post injury and ongoing neurobiological recovery to susceptibility to rmTBI, here we report novel evidence that the inflammatory response at the time of a second mTBI may be associated with susceptibility to rmTBI.

## Supplementary Material

fcag019_Supplementary_Data

## Data Availability

Data will be made available upon request to corresponding author.
