# Supplementary material for: Investigating associations between serum inflammatory cytokines at the time of second mild traumatic brain injury with acute neurological signs, axonal injury and behavioural outcomes in male Sprague–Dawley rats
Source: Brain Commun. 2026 Feb 2;8(1):fcag019. doi: 10.1093/braincomms/fcag019 (PMC12887899; doi:10.1093/braincomms/fcag019)
Supplement: fcag019_Supplementary_Data [file fcag019_supplementary_data.docx]

|  | Plate 1 | | Plate 2 | |  |
| --- | --- | --- | --- | --- | --- |
|  | LLOD (pg/mL) | Intraplate CV (%) | LLOD (pg/mL) | Intraplate CV (%) | Interplate CV (%) |
| IFN-$\gamma$ | 0.419 | 14.59 | 0.188 | 13.98 | 13.8 |
| IL-1$\beta$ | 1.05 | 30.88 | 0.586 | 59.27 | N/A |
| IL-4 | 0.206 | 4.47 | 0.172 | 5.61 | 21.2 |
| IL-5 | 3.23 | 28.46 | 2.48 | N/A | N/A |
| IL-6 | 0.546 | 6.11 | 0.536 | 5.2 | 15.9 |
| IL-10 | 0.569 | 5.36 | 0.605 | 6.41 | 14.4 |
| IL-13 | 0.491 | 6.03 | 0.383 | 6.03 | 5.9 |
| KC/GRO | 0.175 | 6.56 | 0.0479 | 6.83 | 11.8 |
| TNF-$\alpha$ | 0.0879 | 7.65 | 0.0719 | 6.21 | 17.2 |

**Supplementary Table 1**. Lower limit of detection (LLOD) in pg/mL, intraplate coefficient of variance (CV), and interplate coefficient of variance (CV) for each measured inflammatory cytokine. Interplate CV was measured using two samples ran in duplicate on both plates used for inflammatory biomarker analysis.

|  | Sham | 1d | 3d | 7d | 14d |
| --- | --- | --- | --- | --- | --- |
| IFN-$\gamma$ | 19 | 4 | 10 | 13 | 11 |
| IL-4 | 24 | 7 | 11 | 13 | 12 |
| IL-6 | 24 | 7 | 11 | 13 | 12 |
| IL-10 | 24 | 7 | 11 | 13 | 12 |
| IL-13 | 23 | 7 | 11 | 13 | 12 |
| KC/GRO | 23 | 7 | 11 | 10 | 12 |
| TNF-$\alpha$ | 21 | 7 | 11 | 13 | 12 |

**Supplementary Table 2**. Final sample size included for each cytokine in analyses pertaining to the temporal profile of inflammatory cytokines after a single mTBI/sham.

| Correlation (Spearman) | # of observable neurological signs of TBI after 2^nd^ injury | |
| --- | --- | --- |
|  | *r* | *p* |
| IFN-$\gamma$ | -0.15 | 0.35 |
| IL-10 | -0.05 | 0.76 |
| IL-13 | **-0.41** | **0.0067** |
| IL-4 | 0.07 | 0.68 |
| IL-6 | **-0.57** | **<0.0001** |
| KC/GRO | 0.07 | 0.65 |
| TNF-$\alpha$ | 0.08 | 0.60 |

**Supplementary Table 3**. Spearman correlation results for the analysis of inflammatory levels prior to a second injury relating to observable signs of mTBI immediately following the second injury. Adjusted $\alpha$-value 0.0071. P-values <0.0071 presented in red bolded text.

| Correlation (Spearman) | Rotarod | | Morris Water Maze | | | | Elevated Plus Maze | |
| --- | --- | --- | --- | --- | --- | --- | --- | --- |
|  | PID1 relative to baseline | | Acquisition, PID4 | | Reversal, PID5 | | PID12 | |
|  | *r* | *p* | *r* | *p* | *r* | *p* | *r* | *p* |
| IFN-$\gamma$ | 0.28 | 0.09 | 0.16 | 0.33 | -0.04 | 0.79 | -0.004 | 0.98 |
| IL-10 | 0.05 | 0.74 | 0.29 | 0.06 | -0.04 | 0.79 | 0.00 | 0.98 |
| IL-13 | 0.05 | 0.74 | 0.28 | 0.06 | 0.15 | 0.34 | 0.18 | 0.25 |
| IL-4 | 0.00 | 1.00 | **0.32** | **0.04** | 0.13 | 0.42 | 0.11 | 0.47 |
| IL-6 | 0.10 | 0.51 | 0.07 | 0.64 | 0.08 | 0.62 | 0.10 | 0.53 |
| KC/GRO | 0.05 | 0.77 | -0.09 | 0.55 | -0.02 | 0.91 | 0.14 | 0.39 |
| TNF-$\alpha$ | 0.06 | 0.69 | 0.14 | 0.38 | -0.29 | 0.06 | 0.03 | 0.83 |

**Supplementary Table 4**. Spearman correlation results for analysis of inflammatory cytokines with rotarod 1d times relative to baseline, water maze (acquisition and reversal trials), and elevated plus maze. Adjusted $\alpha$-value 0.0071. P-values <0.05 but >0.0071 presented in bolded text.

| Correlation (Spearman) | Serum neurofilament light (NfL) | | | | | | | | | |
| --- | --- | --- | --- | --- | --- | --- | --- | --- | --- | --- |
|  | 0d | | 3d | | 7d | | 14d | | 28d | |
|  | *r* | *p* | *r* | *p* | *r* | *p* | *r* | *p* | *r* | *p* |
| IFN-$\gamma$ | -0.27 | 0.10 | **-0.35** | **0.03** | **-0.39** | **0.02** | -0.26 | 0.11 | -0.25 | 0.14 |
| IL-10 | -0.03 | 0.85 | -0.03 | 0.85 | -0.02 | 0.92 | 0.00 | 1.00 | -0.21 | 0.17 |
| IL-13 | -0.02 | 0.88 | -0.25 | 0.10 | -0.19 | 0.22 | -0.13 | 0.39 | -0.19 | 0.21 |
| IL-4 | 0.10 | 0.51 | 0.07 | 0.64 | 0.06 | 0.71 | 0.11 | 0.50 | -0.14 | 0.36 |
| IL-6 | -0.16 | 0.32 | **-0.35** | **0.02** | -0.22 | 0.16 | -0.26 | 0.10 | -0.04 | 0.78 |
| KC/GRO | 0.10 | 0.56 | 0.05 | 0.78 | 0.01 | 0.95 | -0.02 | 0.88 | -0.30 | 0.06 |
| TNF-$\alpha$ | 0.13 | 0.42 | 0.11 | 0.49 | 0.09 | 0.56 | 0.19 | 0.23 | -0.10 | 0.53 |

**Supplementary Table 5**. Spearman correlation results for analysis of inflammatory cytokines with NfL levels prior to second injury (i.e., 0d), 3-, 7-, 14-, and 28-days post second injury. Adjusted $\alpha$-value 0.0071. P-values <0.05 but >0.0071 presented in bolded text.

| Correlation  (Pearson or Spearman) | | Left Hemisphere | | | | | | | |
| --- | --- | --- | --- | --- | --- | --- | --- | --- | --- |
|  |  | Corpus Callosum | | Internal Capsule | | External Capsule | | Fimbria | |
|  |  | *r* | *p* | *r* | *p* | *r* | *p* | *r* | *p* |
| IFN-$\gamma$ | FA | 0.28 | 0.09 | 0.21 | 0.20 | 0.13 | 0.45 | 0.16 | 0.35 |
|  | ADC | 0.05 | 0.77 | 0.02 | 0.89 | 0.14 | 0.39 | 0.21 | 0.21 |
|  | RD | -0.05 | 0.77 | -0.07 | 0.69 | 0.07 | 0.68 | 0.06 | 0.74 |
|  | AD | 0.31 | 0.06 | 0.16 | 0.34 | 0.25 | 0.13 | 0.32 | 0.05 |
| IL-10 | FA | -0.02 | 0.88 | -0.02 | 0.91 | -0.03 | 0.87 | -0.04 | 0.81 |
|  | ADC | -0.08 | 0.63 | -0.03 | 0.83 | 0.07 | 0.67 | -0.02 | 0.90 |
|  | RD | -0.10 | 0.54 | -0.11 | 0.48 | -0.02 | 0.88 | -0.03 | 0.84 |
|  | AD | -0.04 | 0.81 | -0.02 | 0.88 | 0.05 | 0.73 | 0.10 | 0.54 |
| IL-13 | FA | 0.04 | 0.82 | -0.09 | 0.58 | -0.16 | 0.32 | 0.07 | 0.68 |
|  | ADC | 0.08 | 0.60 | 0.02 | 0.91 | 0.24 | 0.13 | 0.03 | 0.83 |
|  | RD | 0.02 | 0.92 | -0.01 | 0.93 | 0.19 | 0.23 | -0.08 | 0.60 |
|  | AD | 0.19 | 0.23 | 0.02 | 0.88 | 0.21 | 0.18 | 0.18 | 0.26 |
| IL-4 | FA | -0.16 | 0.32 | -0.08 | 0.60 | -0.11 | 0.47 | -0.02 | 0.88 |
|  | ADC | -0.15 | 0.36 | -0.07 | 0.64 | 0.00 | 0.98 | -0.14 | 0.36 |
|  | RD | -0.14 | 0.37 | -0.10 | 0.54 | -0.07 | 0.64 | -0.14 | 0.38 |
|  | AD | -0.11 | 0.50 | -0.08 | 0.62 | -0.03 | 0.84 | -0.06 | 0.73 |
| IL-6 | FA | 0.09 | 0.56 | -0.08 | 0.61 | -0.15 | 0.35 | 0.18 | 0.25 |
|  | ADC | 0.28 | 0.07 | 0.20 | 0.21 | **0.36** | **0.02** | 0.22 | 0.17 |
|  | RD | 0.17 | 0.30 | 0.19 | 0.22 | **0.33** | **0.03** | 0.06 | 0.72 |
|  | AD | **0.37** | **0.02** | 0.23 | 0.14 | **0.36** | **0.02** | **0.36** | **0.02** |
| KC/GRO | FA | -0.03 | 0.86 | 0.24 | 0.14 | 0.19 | 0.24 | 0.13 | 0.44 |
|  | ADC | -0.19 | 0.25 | **-0.42** | **0.01** | -0.27 | 0.10 | -0.24 | 0.14 |
|  | RD | -0.15 | 0.35 | **-0.44** | **0.006** | -0.20 | 0.23 | -0.27 | 0.10 |
|  | AD | -0.19 | 0.26 | **-0.37** | **0.02** | -0.30 | 0.06 | -0.23 | 0.16 |
| TNF-$\alpha$ | FA | -0.04 | 0.82 | 0.01 | 0.94 | 0.09 | 0.58 | 0.12 | 0.44 |
|  | ADC | -0.05 | 0.77 | -0.19 | 0.23 | -0.05 | 0.76 | -0.04 | 0.82 |
|  | RD | -0.05 | 0.75 | -0.22 | 0.15 | -0.08 | 0.62 | -0.09 | 0.58 |
|  | AD | 0.00 | 0.98 | -0.14 | 0.36 | -0.03 | 0.87 | 0.00 | 0.98 |

**Supplementary Table 6**. Correlation results (Pearson or Spearman, where appropriate) for analysis of inflammatory cytokines levels prior to second injury with fractional anisotropy (FA), apparent diffusion coefficient (ADC), radial diffusivity (RD), and axial diffusivity (AD) in the left hemisphere (ipsilateral to injury site) 28-days post second injury. Adjusted $\alpha$-value of 0.0009. P-values <0.05 but >0.0009 presented in bolded text.

| Correlation  (Pearson or Spearman) | | Right Hemisphere | | | | | | | |
| --- | --- | --- | --- | --- | --- | --- | --- | --- | --- |
|  |  | Corpus Callosum | | Internal Capsule | | External Capsule | | Fimbria | |
|  |  | *r* | *p* | *r* | *p* | *r* | *p* | *r* | *p* |
| IFN-$\gamma$ | FA | 0.17 | 0.31 | 0.17 | 0.32 | 0.17 | 0.29 | 0.02 | 0.91 |
|  | ADC | 0.14 | 0.39 | 0.05 | 0.77 | 0.15 | 0.37 | 0.28 | 0.09 |
|  | RD | 0.10 | 0.56 | -0.01 | 0.93 | 0.08 | 0.63 | 0.19 | 0.25 |
|  | AD | 0.29 | 0.08 | 0.15 | 0.37 | 0.23 | 0.16 | **0.34** | **0.04** |
| IL-10 | FA | -0.16 | 0.32 | -0.04 | 0.79 | -0.11 | 0.48 | -0.13 | 0.41 |
|  | ADC | 0.08 | 0.62 | 0.01 | 0.95 | 0.09 | 0.57 | 0.11 | 0.49 |
|  | RD | 0.06 | 0.70 | -0.04 | 0.79 | 0.02 | 0.89 | 0.12 | 0.45 |
|  | AD | 0.00 | 0.99 | -0.01 | 0.97 | 0.06 | 0.71 | 0.18 | 0.25 |
| IL-13 | FA | -0.08 | 0.61 | -0.20 | 0.20 | -0.21 | 0.18 | -0.17 | 0.29 |
|  | ADC | 0.18 | 0.24 | 0.15 | 0.34 | 0.29 | 0.06 | 0.27 | 0.09 |
|  | RD | 0.21 | 0.19 | 0.13 | 0.40 | 0.24 | 0.13 | 0.25 | 0.11 |
|  | AD | 0.16 | 0.32 | 0.11 | 0.49 | 0.27 | 0.08 | **0.32** | **0.04** |
| IL-4 | FA | -0.28 | 0.07 | -0.13 | 0.41 | -0.17 | 0.27 | -0.14 | 0.36 |
|  | ADC | 0.01 | 0.97 | 0.03 | 0.86 | 0.05 | 0.74 | -0.01 | 0.97 |
|  | RD | -0.01 | 0.93 | 0.01 | 0.93 | -0.01 | 0.97 | 0.03 | 0.85 |
|  | AD | -0.08 | 0.62 | -0.01 | 0.96 | 0.00 | 0.98 | 0.01 | 0.93 |
| IL-6 | FA | 0.07 | 0.66 | -0.16 | 0.31 | -0.13 | 0.42 | 0.06 | 0.69 |
|  | ADC | **0.33** | **0.03** | 0.30 | 0.05 | **0.39** | **0.01** | **0.41** | **0.0069** |
|  | RD | 0.24 | 0.12 | 0.26 | 0.10 | **0.35** | **0.02** | 0.30 | 0.05 |
|  | AD | **0.36** | **0.02** | **0.32** | **0.04** | **0.41** | **0.0068** | **0.45** | **0.003** |
| KC/GRO | FA | 0.17 | 0.29 | 0.22 | 0.18 | 0.19 | 0.24 | -0.19 | 0.25 |
|  | ADC | -0.23 | 0.16 | **-0.40** | **0.01** | -0.27 | 0.10 | -0.20 | 0.22 |
|  | RD | -0.19 | 0.26 | **-0.40** | **0.01** | -0.20 | 0.22 | -0.25 | 0.13 |
|  | AD | -0.22 | 0.18 | **-0.36** | **0.02** | -0.28 | 0.09 | -0.13 | 0.43 |
| TNF-$\alpha$ | FA | -0.05 | 0.77 | 0.04 | 0.80 | 0.00 | 0.99 | 0.06 | 0.70 |
|  | ADC | -0.11 | 0.50 | -0.10 | 0.51 | 0.02 | 0.91 | -0.01 | 0.95 |
|  | RD | -0.05 | 0.78 | -0.15 | 0.36 | -0.02 | 0.90 | -0.01 | 0.95 |
|  | AD | -0.11 | 0.50 | -0.07 | 0.68 | 0.03 | 0.86 | 0.02 | 0.89 |

**Supplementary Table 7**. Correlation results (Pearson or Spearman, where appropriate) for analysis of inflammatory cytokines levels prior to second injury with fractional anisotropy (FA), apparent diffusion coefficient (ADC), radial diffusivity (RD), and axial diffusivity (AD) in the right hemisphere (contralateral to injury site) 28-days post second injury. Adjusted $\alpha$-value of 0.0009. P-values <0.05 but >0.0009 presented in bolded text.
